# The miR-130 family promotes cell migration and invasion in bladder cancer through FAK and Akt phosphorylation by regulating PTEN

**DOI:** 10.1038/srep20574

**Published:** 2016-02-03

**Authors:** Hiroshi Egawa, Kentaro Jingushi, Takayuki Hirono, Yuko Ueda, Kaori Kitae, Wataru Nakata, Kazutoshi Fujita, Motohide Uemura, Norio Nonomura, Kazutake Tsujikawa

**Affiliations:** 1 Laboratory of Molecular and Cellular Physiology, Graduate School of Pharmaceutical Sciences, Osaka University, Osaka, Japan; 2 Department of Urology, Graduate School of Medicine, Osaka University, Osaka, Japan

## Abstract

Bladder cancer causes an estimated 150,000 deaths per year worldwide. Although 15% of the recurrent bladder cancer becomes an invasive type, currently used targeted therapy for malignant bladder cancer is still not efficient. We focused on the miR-130 family (miR-130b, miR-301a, and miR-301b) that was significantly upregulated in bladder cancer specimens than that of the normal urothelial specimens. We analyzed the functional significance of miR-130 family using a 5637 bladder cancer cell line and revealed that miR-130 family of inhibitors suppressed cell migration and invasion by downregulating focal adhesion kinase (FAK) and Akt phosphorylation. Mechanistic analyses indicate that the miR-130 family directly targets phosphatase and tensin homolog deleted from chromosome 10 (PTEN), resulting in the upregulation of FAK and Akt phosphorylation. In clinical bladder cancer specimens, downregulation of PTEN was found to be closely correlated with miR-130 family expression levels. Overall, the miR-130 family has a crucial role in malignant progression of bladder cancer and thus the miR-130 family could be a promising therapeutic target for invasive bladder cancer.

Bladder cancer is a major malignancy worldwide with an estimated 380,000 new cases resulting in 150,000 deaths annually^1^. Although 55–60% of primary bladder cancer is superficial, about 50% cases relapse intravesically after surgical removal, and approximately 15–40% of the recurrent bladder cancer becomes invasive and exhibits distant metastasis^2^,^3^. Radical cystectomy has been regarded as the first choice treatment for muscle-invasive bladder cancer, even though it lowers quality of life (QOL) for patients^4^. Even in those muscle-invasive bladder cancer patients who receive optimal treatment with chemotherapy and surgery, the 5-year survival rate is only 60% due to distant recurrence^5^,^6^. Limited therapeutic options for invasive bladder cancer have resulted in a median survival of 15 months for patients with metastatic disease^7^. Therefore, treatment modalities with improved clinical outcomes are urgently required, necessitating the search for novel therapeutic targets for advanced bladder cancer.

MicroRNA (miRNA) is a small noncoding RNA molecule of 20–25 nucleotides, which regulates gene expression through translational repression or mRNA degradation. *In silico* genome-wide analyses have predicted that >60% of all mammalian protein-coding genes can be regulated by miRNAs^8^,^9^. MiRNA can therefore modulate various cellular processes including cell growth, migration or invasion, consequently gaining attention as attractive therapeutic targets for cancer treatment^10^,^11^,^12^ Recently, several studies showed that targeting miRNAs known as miRNA cluster (e.g. miR-17–92 cluster, miRNA-23b/27b/24 cluster)^13^,^14^,^15^ or miRNA family sharing a common seed sequence (e.g. miR-34 family, miR-200 family)^16^,^17^, significantly affected tumour progression. Several reports have shown the impact of each miRNA of the miR-130 family in cancer progression^18^,^19^,^20^, but the comprehensive effect of the miR-130 family molecules on tumour progression, including bladder cancer has not been analyzed. The miR-130 family is composed of miR-130b, miR-301a, and miR-301b, which share a common seed sequence.

Previous studies suggest that the development of noninvasive and invasive bladder cancer occurs through two separate pathways with distinct pathobiology. Early stage bladder cancer (pTa or pT1) is commonly linked to activating mutations of the *FGFR3, HRAS* or *PI3K* genes^21^,^22^,^23^, while advanced stage bladder cancer (≥pT2) is linked to mutations in the tumour suppressor genes *TP53, RB*, and *PTEN*, which can act as a negative regulators of the PI3K/Akt signaling pathway^24^,^25^. Additionally, an integrated study of 131 high grade muscle-invasive bladder cancer samples has revealed dysregulation of the PI3K/Akt signaling pathway in 72% of cases^26^. Collectively, the data suggest that the PI3K/Akt pathway is critical to progression of bladder cancer.

We show here for the first time that the miR-130 family is upregulated in bladder cancer clinical specimens and coordinately promotes bladder cancer cell migration and invasion through the phosphorylation of focal adhesion kinase (FAK) as well as Akt by regulating PTEN expression or sub-cellular localization.

## Results

### The miR-130 family (miR-130b, miR-301a and miR-301b) was significantly upregulated in bladder cancer specimens

**Figure 1:**
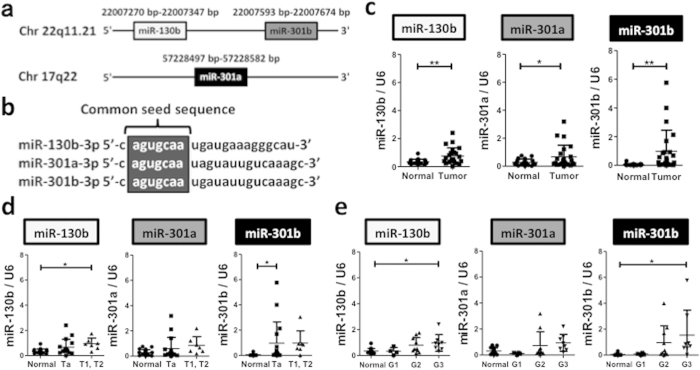
The miR-130 family is significantly upregulated in bladder cancer specimens. (**a**) Genomic localization of the miR-130 family. (**b**) The miR-130 family has a common seed sequence. The expression of miR-130 family was compared between normal and tumour tissues (**c**) among pathological tumour stages (**d**), and among pathological tumour grades (**e**). Data are mean ± S.D. *p < 0.05; **p < 0.01.

We recently found by miRNA microarray analysis, that the miR-130 family molecules including miR-130b, miR-301a and miR-301b, which have a common seed sequence (Fig. 1a,b) were significantly upregulated in invasive renal pelvis and ureter carcinoma, compared to normal upper tract urothelium and their non-invasive counterparts (unpublished data). Since the bladder is also lined with a layer of urothelium, we investigated the expression of the miR-130 family molecules in bladder cancer. We performed quantitative real-time PCR (qRT-PCR) using 19 normal upper tract urothelium tissue samples and 23 bladder cancer tissue samples (Table 1), and found that the miR-130 family was also upregulated in bladder cancer compared to normal upper tract urothelium (Fig. 1c). Although miR-301a showed no significant difference in expression among the different pathologic stages and grades, the expression of miR-130b and miR301b was higher in advanced bladder cancer (Fig. 1d,e).

### The miR-130 family promotes invasion and migration of bladder cancer cells

**Figure 2:**
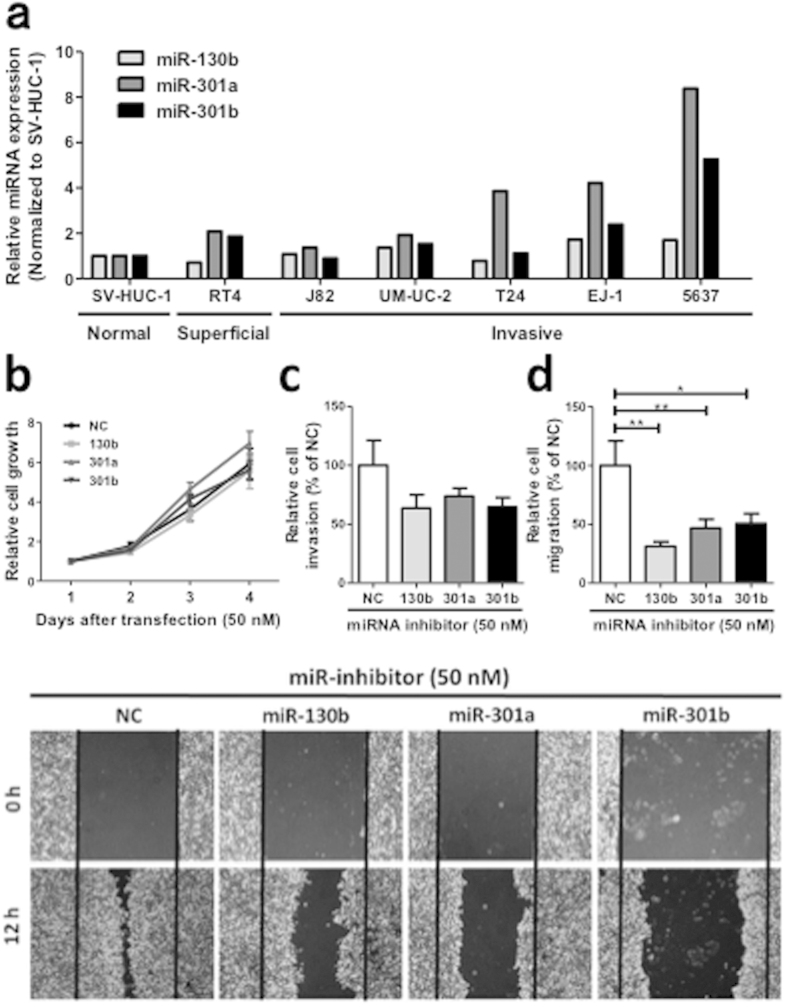
Inhibition of the miR-130 family suppresses 5637 cell invasion and migration. (**a**) Expression of the miR-130 family molecules in bladder cancer cell lines was measured by qRT-PCR. (**b**) The effect of miR-130 family inhibitors on cell growth was measured by a WST-1 assay. (**c**) Invasion assay was performed by xCELLigence real-time cell monitoring system 72 h after transfection. (**d**) Cell migration was estimated by a wound healing assay. The wound was formed by scraping 60 h after transfection and then relative cell migration was measured after 12 h. In all the experiments, 50 nM miRIDIAN hairpin miRNA inhibitors were transfected in 5637 cells. Data are mean ± S.D. of four (**a**) or three (**c,d**) independent experiments. *p < 0.05; **p < 0.01.

**Figure 3:**
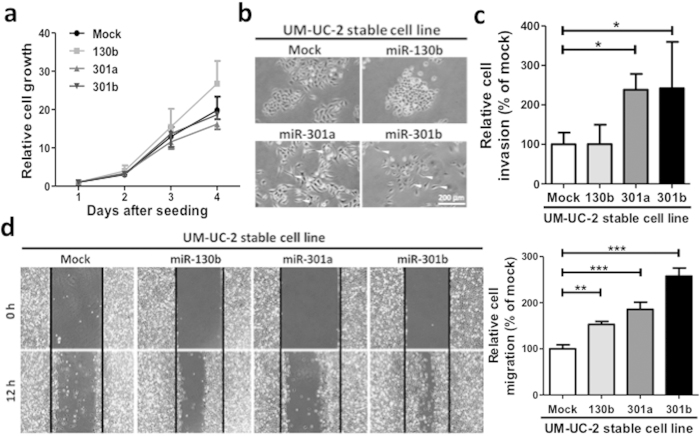
Stable expression of the miR-130 family promotes UM-UC-2 cell invasion and migration. (**a**) Relative cell growth was measured by WST-1 assay (n = 4). (**b**) Characteristic morphologic features of UM-UC-2 cells stably miR-130 family are shown. (**c**) A cell invasion assay was examined. (**d**) Relative cell migration was examined by a wound healing assay. Data are mean ± S.D. of five (**c**) or three (**d**) experiments. **p < 0.01; ***p < 0.001.

To investigate the function of the miR-130 family in bladder cancer cells, we first measured the expression level of the miR-130 family molecules in bladder cancer cell lines. In seven bladder cancer cell lines examined, the highest expression of the miR-130 family was found in 5637 cells (Fig. 2a). Therefore, we used 5637 cells for performing functional analysis of the miR-130 family using miR-130 family inhibitors. The inhibitory effect of the miR-130 family inhibitors was confirmed by luciferase vectors with a miR-130 family molecule-binding site (Supplementary Fig. 1a) in their 3′-UTR. Then, we examined the effect of miR-130 family inhibitors on cell growth, migration, and invasion in 5637 cells. Although they did not significantly affect cell growth (Fig. 2b), the miR-130 family inhibitors reduced invasion (Fig. 2c) and significantly inhibited migration (Fig. 2d and lower panels) of 5637 cells. Next, we established stable cell lines expressing miR-130 family molecules using UM-UC-2 cells, which show a lower miR-130 family expression level (Fig. 2a). The expression levels of miR-130b, miR-301a, and miR-301b were determined by qRT-PCR and observed to be 13.8- and 5.7-, and 14.3-fold higher respectively, compared to a control cell line (mock vector transfection) (Supplementary Fig. 1b). A WST-1 assay showed that overexpression of miR-130 family had no significant effect on UM-UC-2 cell growth (Fig. 3a). However, overexpression of miR-301a and miR-301b induced mesenchymal-like cell morphology (Fig. 3b), and the cell invasion and migration activities were significantly upregulated compared to mock vector-transfected UM-UC-2 cells (Fig. 3c,d). Another clone of UM-UC-2 cells stably overexpressing miR-130 family molecules, also exhibited similar responses while growth was not affected, cell invasion and migration increased compared to mock-transfected cells (Supplementary Fig. 2). Collectively, these results suggest that the miR-130 family molecules regulate cell invasion and migration in bladder cancer cells.

### The miR-130 family promotes stress fiber formation via FAK in bladder cancer cells

**Figure 4:**
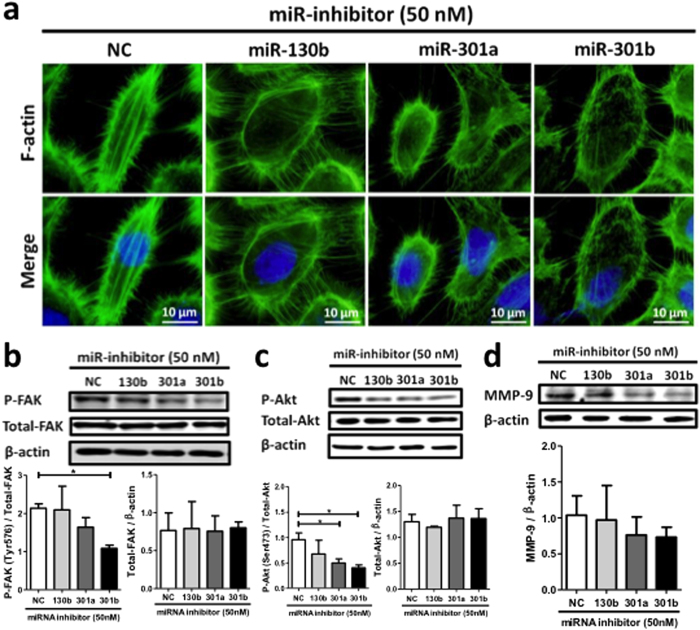
The effects of miR-130 family inhibitors on stress fiber formation, phosphorylation status of FAK and Akt, and MMP-9 expression in 5637 cells. (**a**) Stress fiber formation was observed by F-actin staining with Phalloidin. Phosphorylation status of FAK at Tyr^576^ (**b**) Akt at Ser^473^ (**c**) and MMP-9 expression (**d**) was examined by Western blot analysis. In all experiments, 50 nM miRIDIAN hairpin miRNA inhibitors were transfected in 5637 cells. Data show mean ± S.D. of three independent experiments. *p < 0.05.

The cell invasion and migration activities of bladder cancer cells are dependent on intracellular actin stress fiber formation^27^,^28^,^29^. Stress fibers are connected to the focal adhesion complex consisting of several integrin family molecules and FAK^30^, that plays an important role in cell migration and metastasis. We stained F-actin with fluorescent phalloidin, and analyzed cells by fluorescent microscopy. The miR-130 family inhibitors markedly suppressed stress fiber formation in 5637 cells (Fig. 4a). It is known that the phosphorylation of FAK at Tyr^576^ upregulates stress fiber formation^31^. Therefore we analyzed FAK levels using Western blot. The levels of Tyr^576^-phosphorylated FAK, but not total FAK decreased upon miR-301a/b inhibition (Fig. 4b).

**Figure 5:**
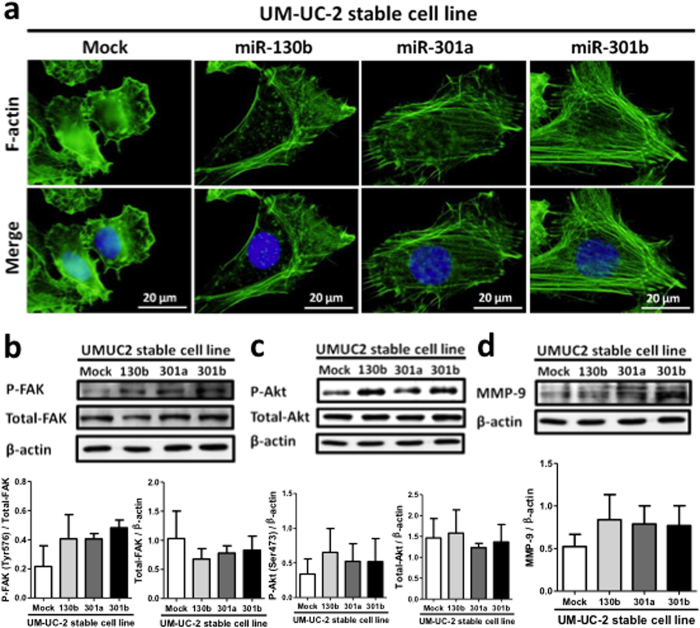
Stress fiber formation, phosphorylation status of FAK and Akt, and MMP-9 expression in the UM-UC-2 cells stably expressing miR-130 family. (**a**) Stress fiber formation was observed by F-actin staining with Phalloidin. Phosphorylation status of FAK at Tyr^576^ (**b**) and Akt at Ser^473^ (**c**) and the protein expression of MMP-9 (**d**) were examined by Western blot analysis. Data show mean ± S.D. of triplicate (b and d) or sextuplicate independent experiments.

We further examined the effect of miR-130 family inhibitors on Akt phosphorylation, which is known as a downstream signaling component of FAK^32^,^33^. The miR-130 family of inhibitors significantly suppressed Akt phosphorylation but not total Akt expression (Fig. 4c). Moreover, the expression of matrix metalloproteinase-9 (MMP-9) protein, a key factor of bladder cancer invasiveness regulated by the PI3K/Akt signaling pathway^34^, decreased in presence of the miR-130 family inhibitors (Fig. 4d). Conversely, stress fiber formation and phosphorylated FAK and Akt levels increased in UM-UC-2 cells stably overexpressing miR-130 family molecules (Fig. 5a–c). Moreover, MMP-9 protein expression also increased in these wells (Fig. 5d). These results suggest that the highly expressed miR-130 family increases migration and invasion activities of bladder cancer cells via FAK activation and subsequent upregulation of Akt signaling pathway.

### The miR-130 family downregulates the protein expression of PTEN in bladder cancer cells

**Figure 6:**
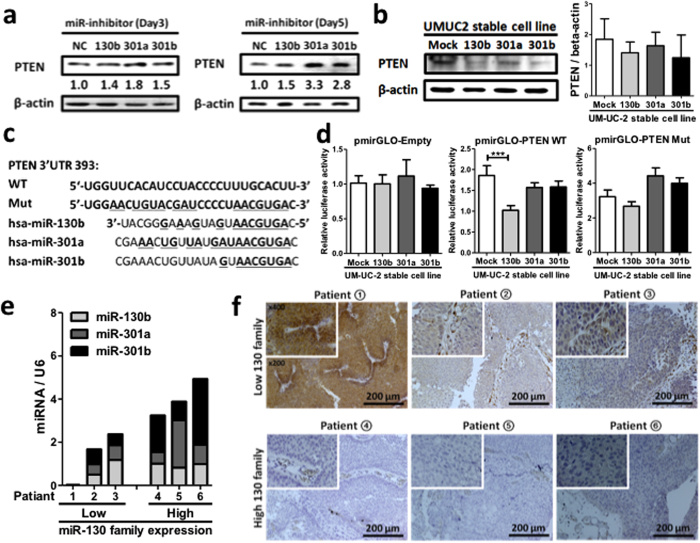
The miR-130 family regulates PTEN protein expression. Western blot analysis of PTEN in miRIDIAN hairpin inhibitor-transfected 5637 cells on days 3 and 5. (**a**), or UM-UC-2 cells stably expressing miR-130 family (**b**). (**c**) A schematic model of predicted miR-130 family binding sequences, and its mutant sequence within the 3′-UTR of human *PTEN* gene. The number indicates the nucleotide position of the predicted miR-130 family binding site from the start of the *PTEN* 3′-UTR. (**d**) A dual luciferase reporter assay was performed with UM-UC-2 cells stably expressing miR-130 family. The cells were transfected with a reporter plasmid containing predicted miR-130 family binding site in the *PTEN* 3′-UTR. Results of MiR-130 family expression determined by qRT-PCR (**e**), and PTEN expression determined by PTEN-immunohistochemical staining (**f**), in bladder cancer clinical samples are shown. All staining images depict a magnified image in the upper left region of the images. Data show mean ± S.D. of three (**a**,**b**), or five independent (**d**) experiments. ***p < 0.001.

To identify a target of the miR-130 family in bladder cancer cells, we utilized target prediction programs (miRBase and microRNA.org), and focused on PTEN as a potential target, since it is known as a tumor suppressor in various cancers and as a negative regulator of the PI3K/Akt signaling pathway^35^,^36^. The PTEN protein levels increased when miR-301a and miR-301b were inhibited (Fig. 6a). On the other hand, the effect of miR-130b inhibitor on the PTEN protein expression was weak due to the low expression of miR-130b in 5637 cells. On the other hand, overexpression of the miR-130 family tended to decrease PTEN expression in UM-UC-2 cells (Fig. 6b). To confirm whether PTEN was a direct target of the miR-130 family, we constructed luciferase reporter vectors carrying the human PTEN 3′-UTR containing either the predicted wild-type, or mutated binding sites for miR-130 family (Fig. 6c). The UM-UC-2 cells stably overexpressing miR-130 family molecules, were transfected with the modified luciferase reporter vectors and a dual-luciferase reporter assay was performed. Stable expression of the miR-130b significantly decreased the luciferase activity in UM-UC-2 cells transfected with the vector containing PTEN wild-type 3′-UTR, but no decrease was detectable when the cells were transfected with the mock vector, or the vector with mutant PTEN 3′-UTR (Fig. 6d). Weak but similar effects were detected in UM-UC-2 cells stably expressing miR-301a/b. From these results, we deduced that miR-130b directly targets PTEN, while miR-301a/b might suppress PTEN expression via an indirect mechanism in bladder cancer cells. To examine the relationship between the expression of miR-130 family and PTEN suppression in clinical samples, we performed immunohistochemistry with anti-PTEN antibody, on formalin-fixed, paraffin-embedded bladder cancer specimens. First, we validated the miR-130 family expression by qRT-PCR in twelve bladder cancer clinical samples, and used the histopathological specimens derived from the samples showing the three highest or lowest expression levels of the miR-130 family for immunohistochemical analysis. As shown in Fig. 6e,f, PTEN was strongly expressed in the bladder cancer specimens with lower miR-130 family expression, whereas almost no expression was observed in specimens exhibiting higher miR-130 family expression. Collectively, the data strongly indicate that the miR-130 family negatively regulates PTEN protein expression in bladder cancer cells, promoting their migration and invasion properties.

## Discussion

Over the past two decades, there has been no significant improvement of therapeutic agents for locally advanced or metastatic bladder cancer^37^. Therefore, identification of a novel molecular target for bladder cancer treatment is urgently desired. In the present study, we have identified the miR-130 family members as potential molecular targets possessing a novel action mechanism, for bladder cancer treatment.

The integrated analysis of 131 muscle-invasive bladder cancer specimens reveals that dysregulation of PI3K/Akt pathway is detected in 72% of the tumours^26^, hence regulatory elements of the PI3K/Akt pathway have received much attention as potential therapeutic targets 38. PTEN is a negative regulator of the PI3K/Akt signaling pathway^35^,^39^, and shows loss of heterozygosity (LOH) in muscle-invasive bladder cancers^40^. However, the LOH in the PTEN region was detected in only 24.5% of the bladder cancer specimens. Therefore, the presence of alternative mechanisms of PTEN regulation is predicted in bladder cancer. We report here that the miR-130 family molecules are novel negative regulators of PTEN expression in bladder cancer. In the “Two-pathway model of bladder cancer”, inactivation of PTEN is considered a trigger for progression from non-invasive to invasive tumour^25^,^41^,^42^. We confirmed that the forced expression of PTEN reduced Akt phosphorylation as well as the migration of 5637 cells (Supplementary Fig. 3). Therefore, we conclude that the miR-130 family plays a role in bladder cancer progression via activation of PI3K/Akt signaling pathway by suppressing PTEN expression.

Although our data showed that miR-130 family molecules upregulated bladder cancer cell migration and invasion, the suppression mechanisms of PTEN expression seem to be different between miR-130b and miR-301a/b. It is reported that the membrane localization of PTEN is important for its phosphatase activity and protein stability^35^,^43^,^44^. We observed that the miR-130 family inhibited the membrane localization of PTEN in a bladder cancer cell line (Supplementary Fig. 4). Membrane localization of PTEN is mainly maintained by the interaction with membrane-associated proteins or the phosphorylation of PTEN C-terminal tail such as Ser^380^ 44. A membrane-associated guanylate kinase family protein with multiple PDZ domains (MAGI2) is well known as a membrane-associated protein that can interact with PTEN to promote stability and subsequent phosphatase activity^45^. Although MAGI2 has a predicted miR-130 family target sequence in the 3′-UTR, the stable expression of the miR-130 family molecules had no significant effect on MAGI2 luciferase assay (Supplementary Fig. 5). The conformational changes induced by phosphorylation of PTEN C-terminal tail at Ser^380^ suppresses PTEN activation, by preventing its recruitment to the cell membrane^46^. As shown in Supplementary Fig. 4c, the stable expression of the miR-130 family molecules upregulated PTEN phosphorylation at Ser^380^ consistent with the increased Akt phosphorylation levels (Fig. 5c), indicating miR-130 family suppresses PTEN activation. The glioma tumour suppressor candidate region 2 (GLTSCR2, also known as PICT-1) and RhoA-associated kinase 1/2 (ROCK1/2) are shown to promote its phosphorylation at Ser^380^ 35,47. ROCK1/2 are well-validated downstream signaling components of RhoA^48^, and pharmacological inhibition of tyrosine-protein phosphatase non-receptor type 11 (PTPN11) stimulates RhoA activity^49^. Importantly, PTPN11 harbors a predicted miR-130 family target site in its 3′-UTR, and the dual-luciferase assay and western blot analysis revealed that miR-130b and miR-301b directly targeted PTPN11 (Supplementary Fig. 5b,c). Although the PTEN localization mechanism regulated by miR-301a is under investigation, miR-301b possibly regulates PTEN localization by targeting PTPN11 and subsequent RhoA-ROCK1/2 activation.

Although all the miR-130 family molecules were upregulated in bladder cancer specimens, it is very interesting that functional differences exist among the family members. The miR-130 family molecules possess slightly different sequences, in spite of their common seed region (Fig. 1b), resulting in regulation of different target genes. We observed that miR-130b had little effect on bladder cell migration and invasion. However, miR-130b is known to promote other oncogenic phenotypes such as stemness, drug resistance^18^,^50^, and angiogenesis^51^, by targeting tumor protein p53-inducible nuclear protein 1 (TP53INP), and Peroxisome Proliferator-Activated Receptor ɤ (PPAR ɤ), respectively. Therefore, miR-130b may contribute to bladder cancer progression by a distinct mechanism from miR-301a/b. As shown in Figs 3 and 5, UM-UC-2 cells overexpressing miR-130b, miR-301a, and especially miR-301b exhibited strikingly high cell motility and invasiveness, and phosphorylation of FAK. These findings further support that PTPN11 is possibly an additional direct target gene of the miR-130 family in bladder cancer cells. Both inhibition and overexpression of miR-301b were confirmed to regulate PTPN11 protein expression, suggesting PTPN11 as a direct target gene of miR-301b in bladder cancer. Importantly, PTPN11 dephosphorylates tyrosine residues in FAK^52^, and functional inhibition of PTPN11 leads to the formation of stress fiber and focal adhesion^49^,^53^. This finding is consistent with the distinct phenotype of the miR-301b-overexpressing UM-UC-2 cells. In addition to these genes, the miR-130 family may have other potential targets that act as tumour suppressors (e.g., TIMP2^54^, TSC1^55^, RUNX3^56^, TP63^20^,^41^,^42^, Smad4^57^, and CDKN1A^58^). Collectively, the miR-130 family might modulate cellular signaling pathways of bladder cancer cells by regulating various target genes, including PTEN and PTPN11.

Previous findings about the function of miRNA family in bladder cancer were related only to the tumour suppressor miR-200 family, whose inhibition induces epithelial-mesenchymal transition (EMT), resulting in the promotion of mesenchymal phenotype^17^. Therefore, our findings on the miR-130 family are the first report that clarifies the role of oncomiR family in bladder cancer. Given the clinical application of tumour suppressor miRNA such as the miR-200 family, restoring miRNA activity by using double-stranded miRNA mimic might be a possible therapeutic strategy. However, double-stranded miRNA mimics can potentially induce a non-specific interferon response though Toll-like receptors^59^. Therefore, inhibition of miRNA function might be a more appropriate therapeutic strategy. Each miRNA typically targets approximately 200 genes^60^,^61^, which in turn, enables modulation of entire pathways in a disease of interest, by targeting disease-associated miRNAs. However, the miRNA-mediated mechanism of translational repression has a relatively weak effect on target genes. Therefore, targeting a particular miRNA family that has overlapping roles in disease might enable wide and powerful inhibition of disease-related signaling pathways. Rottiers *et al*. have reported the effect of pharmacological inhibition of the miR-33 family using seed-targeting 8-mer locked nucleic acid (LNA) in an African green monkey metabolic disease model^62^. Treatment with miR-33 family-targeted LNA resulted in depression of direct miR-33 family target genes and increased the circulating high density lipoprotein (HDL) cholesterol levels more strongly than miR-33a or miR-33b inhibition alone. In case of the miR-130 family molecules, functional overlap in various biological processes is reported^53^,^63^,^64^,^65^, thus simultaneous inhibition of the miR-130 family can be expected to lead to effective therapeutic effects in bladder cancer.

Recently, the pan-cancer oncogenic miRNA superfamily has been identified using the Cancer Genome Atlas (TCGA) pan-cancer data set^66^. The miR-130 family is included in the miRNA superfamily, and is upregulated in several types of cancer such as bladder, breast, lung, head and neck. Furthermore, the expression of miR-130 family members is significantly negatively correlated with the several tumour suppressor genes including PTEN, across 8 tumour types. These findings suggest that the miR-130 family members undergo coordinate regulation in tumours to mediate silencing of tumour suppressor genes in a synergistic manner. Generally, it is well known that bladder cancer shows extensive heterogeneity in clinical behavior. Now it is classified into five major subtypes based on various gene expression signatures^42^. Therefore, targeting the miR-130 family, which can regulate multiple cancer-related signaling pathways, would provide a most effective therapeutic strategy for the bladder cancer treatment.

## Materials and Methods

### Plasmid construction

To construct the miR-130 family or PTEN reporter plasmids, the following oligonucleotides for miR-130 family-putative targeted sequences and *PTEN* were used:

hsa-miR-130b sense 5′-CTAGCGGCCGCTAGTATGCCCTTTCATCATTGCACTGG-3′, antisense 5′-TCGACCAGTGCAATGATGAAAGGGCATACTAGCGGCCGCTAGAGCT-3′, hsa-miR-301a sense 5′-CTAGCGGCCGCTAGTGCTTTGACAATACTATTGCACTGG-3′, antisense 5′-TCGACCAGTGCAATAGTATTGTCAAAGCACTAGCGGCCGCTAGAGCT-3′, hsa-miR-301b sense 5′-CTAGCGGCCGCTAGTGCTTTGACAATATCATTGCACTGG-3′, antisense 5′-TCGACCAGTGCAATGATATTGTCAAAGCACTAGCGGCCGCTAGAGCT-3′, human PTEN 3′-UTR sense 5′-CTAGCGGCCGCTAGTTGGTTCACATCCTACCCCTTTGC-ACTTG-3′, antisense 5′-TCGACAAGTGCAAAGGGGTAGGATGTGAACCAACTAGCGGCCGCTAG-AGCT-3′, human MAGI-2 3′-UTR sense 5′-CTAGCGGCCGCTAGTTCTCCATGACTTCATTTGCACT-TG-3′, antisense 5′-TCGACAAGTGCAAATGAAGTCATGGAGAACTAGCGGCCGCTAGAGCT-3′. human PTPN11 3′-UTR sense 5′-TTGTGAGCTCTATTTTGCAGATTATGGGGA-3′, antisense 5′-TTGTGTCGACCATTTGGCGACCAAAAACAC-3′. human PTPN11 mut 3′-UTR sense 5′-AGTTGACCTAACGTGAGGCATTAAAGAGTC-3′, antisense 5′-GACTCTTTAATGCCTCACGTTAGGTCAACT-3′.

The annealed products were digested with *Sac* I and *Sal* I, and inserted into the pmirGLO dual-luciferase miRNA target expression vector. The primary hsa-miR-130b, hsa-miR-301a, and hsa-miR-301b were cloned from genomic DNA of 5637 cells by RT-PCR using KOD-FX and oligonucleotide primers as follows:

hsa-miR-130b sense 5′-TCGAAAGCTTTACCCAATTCGCTCCCTTCT-3′, antisense 5′-TCGAGGATCCCACCCACCTGATCCTCTGAT-3′, hsa-miR-301a sense 5′-GCGAATTCTCCAAATATGTAACAGAAAGCAACA-3′, antisense 5′-GCGGATCCTTCCTTTCTACATCTATGCATGTTT-3′, hsa-miR-301b sense 5′-GCAAGCTTGGTGTCCTGGGTTCTGAAGACC-3′, antisense 5′-GCGGATCCCAGGCCTGTCTAGAATCTCAAGTT-3′.

The PCR products were digested with HindIII and BamHI (hsa-miR-130b / hsa-miR-301b), or with EcoRI and BamHI (hsa-miR-301a), and inserted into the pmR-ZsGreen1 miRNA expression vector.

### Clinical specimens

Bladder cancer tissue specimens were obtained from patients who had undergone transurethral resection of bladder tumour at the Osaka University Medical Hospital, Japan. Normal urothelial specimens were also obtained from resected tissues of renal pelvis and ureter. Tumours were staged according to the 6th AJCC TNM staging system and graded according to Fuhrman′s nuclear grading system. Prior written and informed consent was obtained from all patients, and the study was approved by the institutional review board of the Osaka University Hospital and the methods were carried out in accordance with the approved guidelines. Clinical and histopathological data related to the clinical samples are presented in Table 1.

### Quantitative real-time PCR (qRT-PCR)

Following operation, the tissue samples were immediately immersed in RNAlater, (Qiagen, Valencia, CA, USA) and stored at −20 °C until RNA extraction. miRNA was purified using the miRNeasy mini Kit (Qiagen). A qRT-PCR was conducted to determine the expression of miR-130 family molecules in normal upper tract urothelium tissue samples and 23 bladder cancer tissue samples by using MiR-X miRNA first-strand synthesis kit (Takara, Shiga, Japan) in duplicate. Thermal cycling conditions included an initial step at 98 °C for 30 seconds and 40 cycles at 95 °C for 2 seconds, and at 63 °C for 5 seconds by using a miR-130 family specific primer as follows: miR-130b: 5′-CAGTGCAATGATGAAAGGGCAT-3′, miR-301a: 5′-CAGTGCAATAGTATTGTCAAAGC-3′, miR-301b: 5′-CAGTGCAATGATATTGTCAA-AGC-3′, and a U6 snRNA-specific primer (Takara) as an internal control.

### Cell culture and transfection

Human bladder cancer cell lines 5637^67^ and UM-UC-2^68^ were cultured in RPMI 1640 medium (Wako, Osaka, Japan) and Dulbecco’s modified Eagle’s medium (DMEM: Wako), respectively, supplemented with 10% heat-inactivated fetal bovine serum (FBS) and 100 mg/L kanamycin at 37 °C under a 5% CO_2_ atmosphere. 5637 and UM-UC-2 were each supplied from Department of Urology, Osaka University (Osaka, Japan) and Nara Medical University (Nara, Japan).

MiRIDIAN miRNA hairpin inhibitors for human hsa-miR-130b-3p (IH-300660-07-0005), hsa-miR-301a-3p (IH-300657-05-0005), hsa-miR-301b-3p (IH-301252-02-0005) and negative control (IN-001005-01-05) were purchased from GE Healthcare (Lafayette, CO, USA). The miRIDIAN miRNA hairpin inhibitors were transfected at a concentration of 50 nmol/L using Lipofectamine RNAiMAX reagent (Life Technologies, Carlsbad, CA, USA). For transfection with plasmid DNA (Supplementary Fig. 3), Lipofectamine 2000 reagent (Life Technologies) was used. These transfection experiments were performed according to the protocol supplied by the manufacturer.

### Water-soluble tetrazolium salt-1 (WST-1) cell proliferation assay

Cell proliferation was examined by a WST-1 cell proliferation assay. MiRIDIAN hairpin inhibitor-transfected 5637 cells were reseeded in a 96-well plate (2 × 10^3^ cells/well) 24 h after transfection, and incubated for indicated times. After incubation for 1 h with WST-1 reagent (Dojindo Laboratories, Osaka, Japan) at 37 °C and 5% CO_2_, the optical density was read at a wavelength of 450/630 nm (measurement/reference) by Model 680 Microplate Reader (Bio-Rad, Hercules, CA, USA).

### Construction of UM-UC-2 cells with stable miR-130 family overexpression

The pri-hsa-miR-130b, pri-hsa-miR-301a, or pri-hsa-miR-301b expression pmR-ZsGreen1 vectors were transfected into UM-UC-2 cells seeded at 5 × 10^4^ cells/well in a 12-well plate using Lipofectamine 2000 as a transfection reagent. After 24 hours of transfection, culture medium was changed to new medium containing 800 μg/mL G418 (Wako) to select for transfectant cells. These cells were subjected to limited dilution cloning in a 96-well plate (0.7 cells/well) to form a single colony, and ZsGreen1-positive colonies were subjected to miRNA purification, and qRT-PCR to confirm expression of miR-130b family members.

### Wound healing assay

5637 cells transfected with miRIDIAN hairpin inhibitor were seeded in a 12 well plate (7.5 × 10^4^ cells/well) and incubated for 72 h. A wound was created in a monolayer of 5637 cells or the UM-UC-2 cells stably expressing miR-130 family at ~90% confluence, by using a sterile 1 mL pipette tip. Cell pictures were recorded at 0 and 12 h after wound creation by using OLYMPUS IX71 fluorescence microscope (Olympus, Tokyo, Japan).

### Cell invasion assay

The Corning tumour invasion system with the 8.0 μm pore size FluoroBlok membrane (Corning, New York, NY, USA) was used to perform the cell invasion assay for the UM-UC-2 cells stably expressing miR-130 family. The cells were seeded in the insert of a 96-well plate (3 × 10^3^ cells/well) in serum-free conditions, and DMEM containing 10% FBS was used as a chemo-attractant in the base plate. Following incubation for 24 h, the cells were labeled with calcein AM (4 μg/mL), and the fluorescence of the invading cells was read at wavelengths of 494/517 nm (Ex/Em) by EnVision Multilabel Reader (PerkinElmer, Waltham, MA, USA). For miRIDIAN hairpin Inhibitor-transfected 5637 cells, the cell invasion was measured by xCELLigence system (Roche, Indianapolis, IN, USA) according to the manufactured protocol.

### Western blotting analysis

The whole cell lysate was separated by sodium dodecyl sulfate (SDS)-polyacrylamide gel electrophoresis (PAGE) and then transferred to a polyvinylidene difluoride (PVDF: Millipore, Billerica, MA, USA) membrane by using the semidry transfer system (Bio-Rad). The membranes were probed with specific antibodies as indicated and then incubated with horseradish peroxidase (HRP)-conjugated antibody against mouse or rabbit immunoglobulin (1:5000, Cell Signaling Technology:CST, Beverly, MA, USA), followed by detection with enhanced chemiluminescence (ECL) Western blotting detection reagents (GE Healthcare). ImageQuant LAS4000 mini system (GE Healthcare) was used as a chemiluminescence detector.

The following antibodies were used for immunological analysis in this study: anti-FAK polyclonal (1:1000, Santa Cruz, sc-557, Santa Cruz, CA, USA), anti-p-FAK^576^ (1:1000, Sigma, SAB4503869, St Louis, MO, USA), anti-p-FAK^576^ (1:1000, Santa Cruz, sc-16563-R), anti-MMP9 (1:1000, CST, #3852S), anti-Akt (1:1000, CST, #C67E7), anti-p-Akt^473^ (1:1000, CST, #D9E), anti-PTEN polyclonal (1:1000, Sigma, SAB4300336: for western blot analysis and immunocytochemistry), anti-PTEN polyclonal (1:1000, CST, #9188: for western blot analysis and immunohistochemistry), anti-Actin polyclonal antibody (1:50000, Sigma, A5316).

### Microscopic observations

For fluorescence microscopy observation of cultured cells, cells were grown on a micro coverglass, fixed by incubating in 4% formaldehyde, and then permeabilized with blocking buffer containing 5% BSA, 0.1% Triton X-100 in phosphate buffered saline (PBS). The permeabilized cells were incubated with the first antibody at 4 °C overnight, followed by fluorochrome-conjugated secondary antibody for 1 h at room temperature. For F-actin staining, the permeabilized cells were incubated with 40 nmol/L Acti-stain 488 Fluorescent Phalloidin (Cytoskeleton, Inc., Denver, USA) at room temperature for 3 h. After that, coverslips were mounted into a slide glass using Dapi Fluoromount-G (SouthernBiotech, Birmingham, AL, USA). Fluorescent images were obtained with DP70 fluorescence microscope (Olympus).

### Dual-luciferase reporter assay

A pmirGLO dual-luciferase miRNA target expression vector was used for the 3′-untranslated region (UTR) luciferase reporter assay (Promega, Madison, WI, USA). The sequences of the inserts are given in “plasmid construction” section. The UM-UC-2 cells stably expressing miR-130 family were transfected with the reporter construct containing the predicted miR-130 family binding site in the PTEN (Fig. 6c) or MAGI2 (Supplementary Fig. 5) 3′-UTR. After 48 h of transfection, the dual-luciferase reporter assay was performed according to the manufacturer′s protocol (Promega). Luciferase activity was determined using a luminometer (Turner Biosystems 20/20 luminometer; Promega).

### Immunohistochemistry

The expression of PTEN was determined by immunohistochemical staining of the paraffin-embedded tissues obtained from bladder cancer patients. Formalin-fixed, paraffin-embedded tissue sections (5 μm in thickness) were deparaffinized and rehydrated. After the slides were steamed for 10 min in instant antigen activation solution H (LSI Medience Corporation, Tokyo, Japan) for antigen retrieval, endogenous peroxidase was blocked using 3% H_2_O_2_. Immunohistochemical staining for PTEN was performed using anti-PTEN antibody (CST, 1:100), and the Envision Detection System (Dako, Tokyo, Japan) was used according to the manufacturer′s instructions. Primary antibodies were incubated overnight at 4 °C and counter-stained with hematoxylin. Images were obtained with Bio-Zero fluorescence microscope (KEYENCE, Osaka, Japan).

### Statistics

Results were expressed as the mean ± S.D. Differences between values were statistically analyzed using a Student′s t-test or one-way ANOVA with Bonferroni post-hoc tests (GraphPad Prism 5.0, GraphPad Software, San Diego, CA, USA). A *p*-value <0.05 was considered statistically significant.

## Additional Information

**How to cite this article**: Egawa, H. *et al*. The miR-130 family promotes cell migration and invasion in bladder cancer through FAK and Akt phosphorylation by regulating PTEN. *Sci. Rep.* **6**, 20574; doi: 10.1038/srep20574 (2016).

## Supplementary Material

Supplementary Information

## Figures and Tables

**Table 1:** Bladder cancer clinical samples used for qPCR analysis and Immunohistochemistry.

	For real-time PCR analysis (validation of miR-130 family expression)	For immunohisto-chemistry
**Age (Year)**		
Mean	72	69
Range	47–86	61–79
**Gender**		
Male	16	5
Female	7	1
**Pathologic stage**		
Ta	16	3
T1	5	2
T2	2	1
**Pathologic grade**		
G1	4	1
G2	10	1
G3	8	4
Unknown	1	
