# Supplementary material for: The miR-130 family promotes cell migration and invasion in bladder cancer through FAK and Akt phosphorylation by regulating PTEN
Source: Sci Rep. 2016 Feb 3;6:20574. doi: 10.1038/srep20574 (PMC4738343; doi:10.1038/srep20574)

The miR-130 family promotes cell migration and invasion in bladder cancer through FAK and Akt phosphorylation by regulating PTEN

Hiroshi Egawa, Kentaro Jingushi, Takayuki Hirono, Yuko Ueda, Kaori Kitae, Wataru Nakata, Kazutoshi Fujita, Motohide Uemura, Norio Nonomura, Kazutake Tsujikawa

Supporting Figure legend

Supplementary Figure 1 Functional verification of miR-130 family hairpin inhibitors and establishment of miR-130 family stable expression cell lines. (a) MiR-130 family reporter vectors containing perfectly matched target sites of miR-130b, miR-301a or miR-301b (50 ng) were cotransfected with 50 nM miRIDIAN hairpin inhibitors or a negative control inhibitor into 5637 cells. Luciferase activity was determined using a dual reporter assay system. Data are presented as mean ± S.D. of more than three independent experiments. *$p < 0.05$, **$p < 0.01$, ***$p < 0.001$. (b) Relative miR-130 family expression in established UM-UC-2 stable cell lines. The expression levels of each miRNAs were determined with qRT-PCR by a ΔΔCT method, and compared with a mock-transfected cell line.

Supplementary Figure 2 Functional analysis of another clone of UM-UC-2 cells stably expressing miR-130 family. (a) Relative miR-130 family expression in another clone of UM-UC-2 cells stably expressing miR-130 family (Clone No. 2). The expression level of each miRNA was determined with qRT-PCR by ΔΔCT method, and compared with a mock-transfected cell line. (b) The established UM-UC-2 cells (clone No. 2) were seeded into a 96-well plate dish, incubated for the indicated times and relative cell proliferation was measured by a WST-1 assay. Data are mean ± S.D. of five independent experiments. (c) Relative cell motility was measured 12 h after a scratch wound was formed in the cell layer. Representative results of cell motility in the scratch wound- healing assay are shown in the lower panels. Data are mean ± S.D. of three independent experiments. *$p < 0.05$, **$p < 0.01$, ***$p < 0.001$.

Supplementary Figure 3 Transient expression of PTEN suppresses 5637 cell migration. One µg pcDNA 3.0 (Empty) or pcDNA 3.0-PTEN (PTEN) vectors were transfected into 5637 cells. The following experiments were performed 72 h after transfection: (a) Western blot analysis of PTEN, Akt, FAK and their phosphorylated forms using lysates of the transfected 5637 cells. (b) A wound healing assay was performed and relative cell migration was shown as mean ± S.D. of triplicate experiments. *p <0 .05. (c) Representative images of cell membrane localization of PTEN in transfected 5637 cells. The cells were stained with anti-PTEN antibody (PTEN) and 4',6-Diamidino-2- phenylindole dihydrochloride (DAPI, Nucleus). (d) The number of cells in which cell membrane localization of PTEN was detectable (indicated by arrowheads in (c)), was counted. Data are mean ± S.D. of triplicate experiments (> 27 cells /view). **p < 0.01.

Supplementary Figure 4 The miR-130 family regulates PTEN localization via its phosphorylation. (a) UM-UC-2 cells stably expressing MiR-130 family were stained with anti-PTEN antibody and DAPI (Nucleus). (b) Representative images of sub-cellular localization of PTEN in miR-130 family-stably expressing UM-UC-2 cells. Cell membrane localization of PTEN is indicated by arrowhead. The number of cells in which cell membrane localization of PTEN was detectable were counted and are shown as mean ± S.D. of triplicate experiments (>34 cells/view). *** $p < 0.001$. (c) The cell lysates were immunoblotted with anti-PTEN and anti-Ser^{380} antibodies, and relative expression of phosphorylated PTEN (P-PTEN) is shown as mean±S.D. of three independent experiments.

Supplementary Figure 5 Identification and validation of an additional miR-130 family target gene in bladder cancer. (a) A schematic model of predicted miR-130 family-binding sites within the 3'-UTR of human MAGI2 gene and the result of dual-luciferase reporter assay for UM-UC-2 cells stably expressing miR-130 family. (b) A schematic model of predicted miR-130 family-binding sites within the 3'-UTR of human PTPN11 gene. A dual-luciferase reporter assay was performed, with pmirGLO-PTPN11 wild-type (WT) or -PTPN11 mutant (Mut) vector, for UM-UC-2 cells stably expressing miR-130 family. (c) The effect of miR-130 family overexpression or inhibition on PTPN11 protein expression was evaluated by western blot analysis. Data shown are mean ± S.D. of five independent experiments (*$p < 0.05$).

Supplementary figure 1

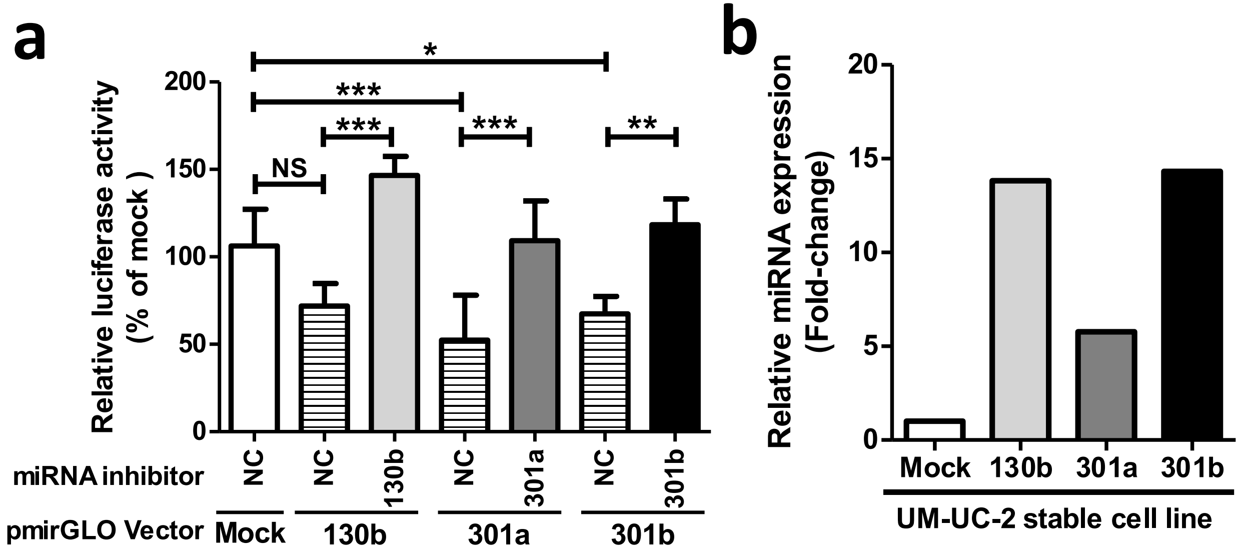

Supplementary figure 2

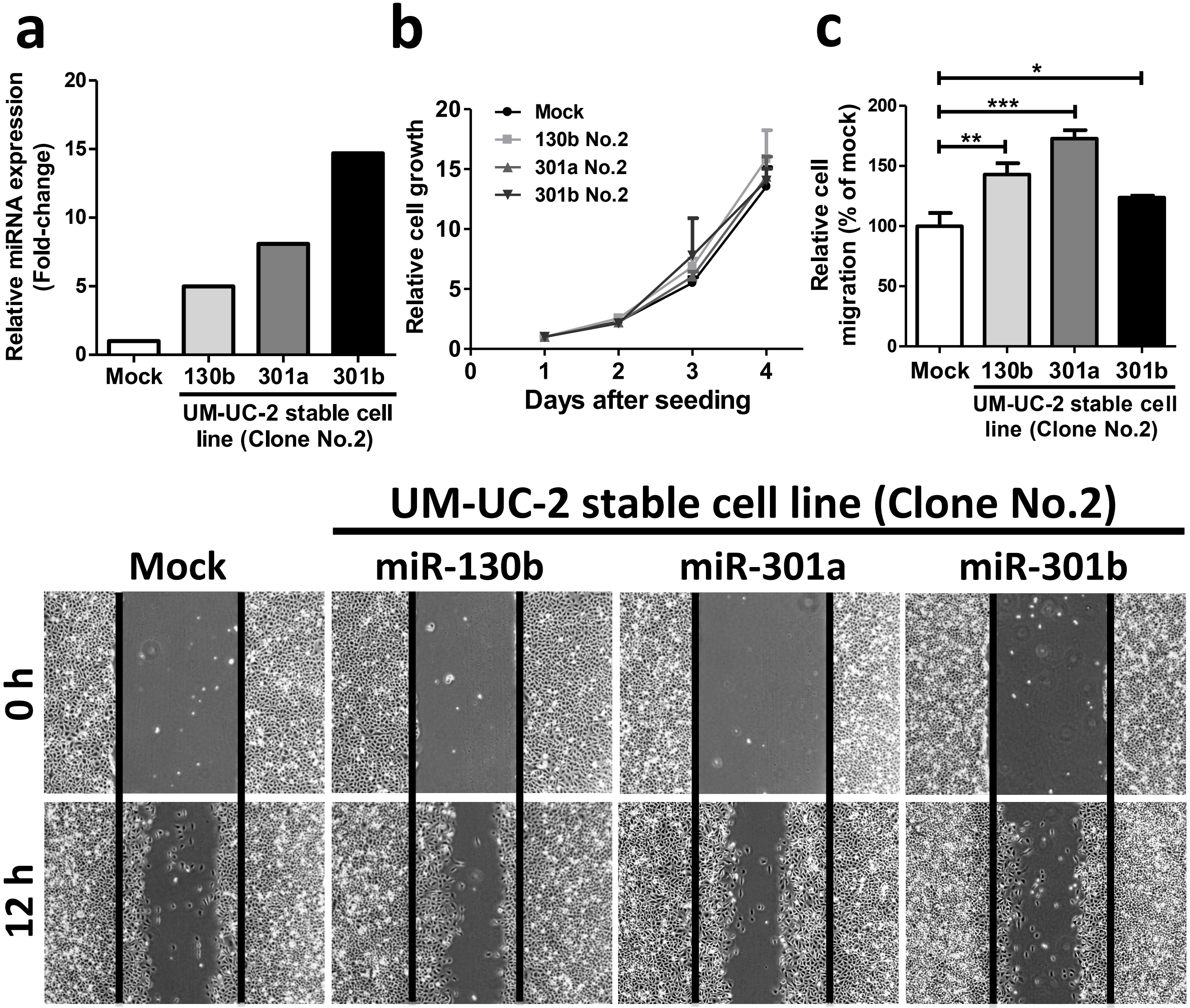

Supplementary figure 3

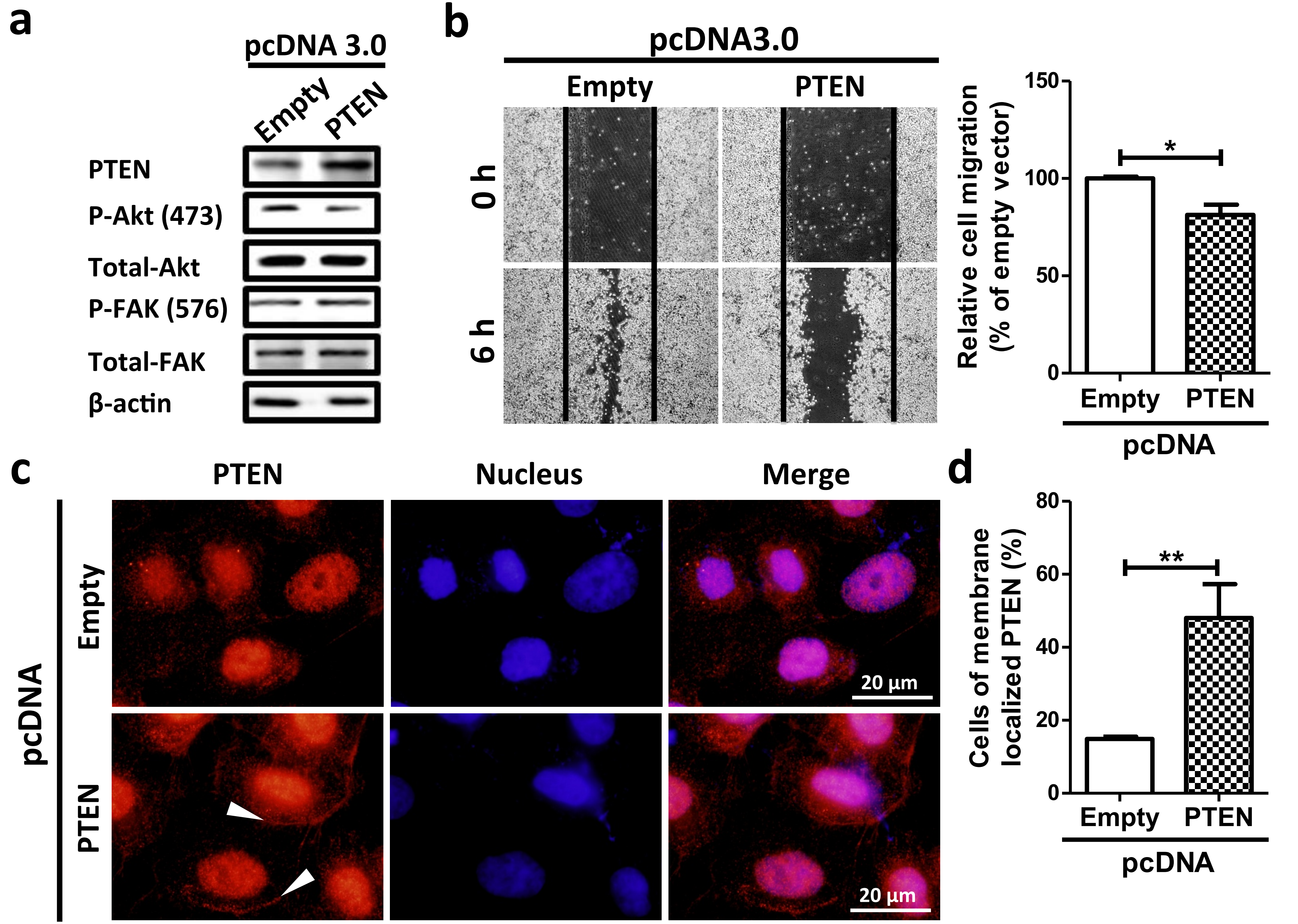

Supplementary figure 4

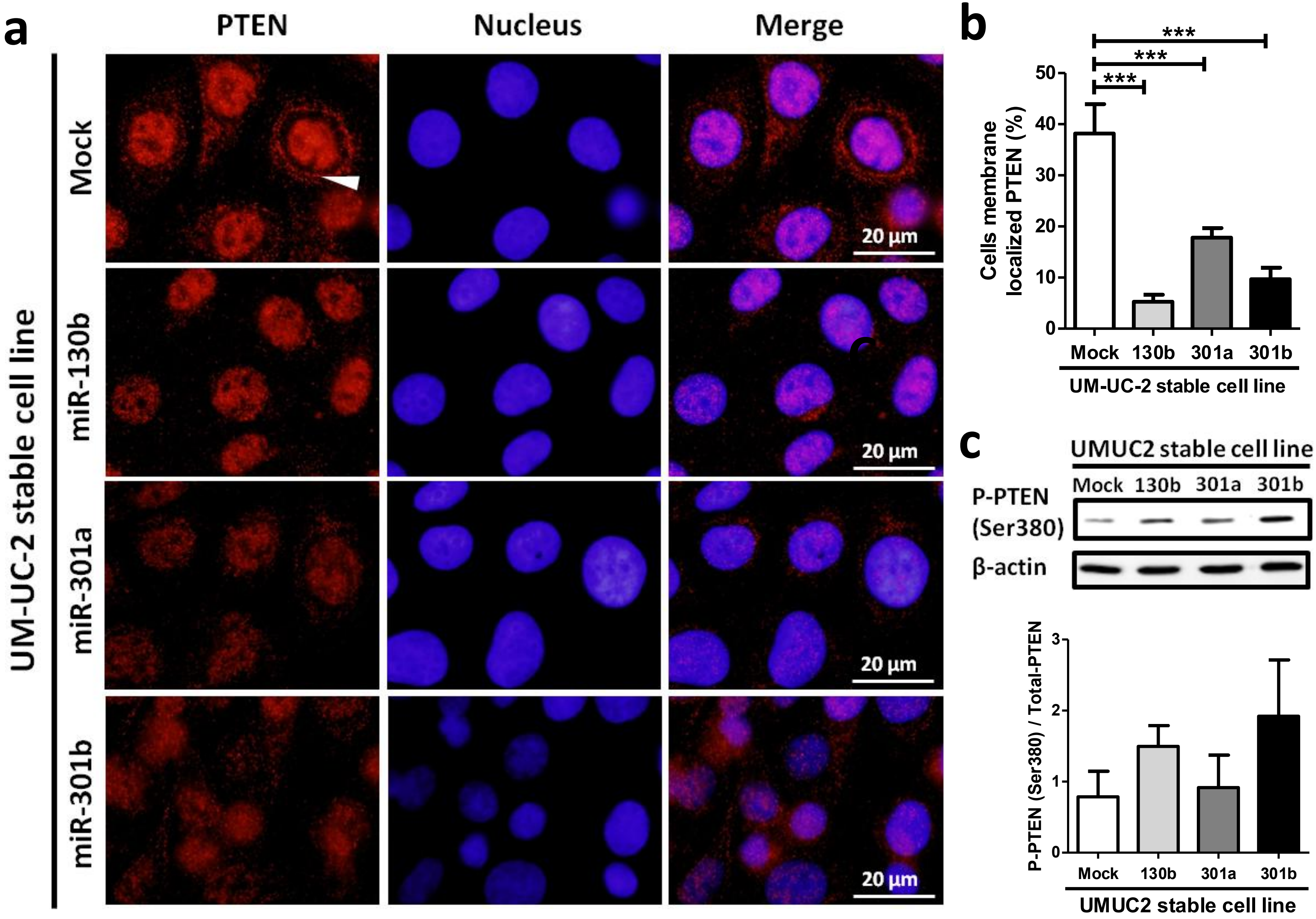

Supplementary figure 5

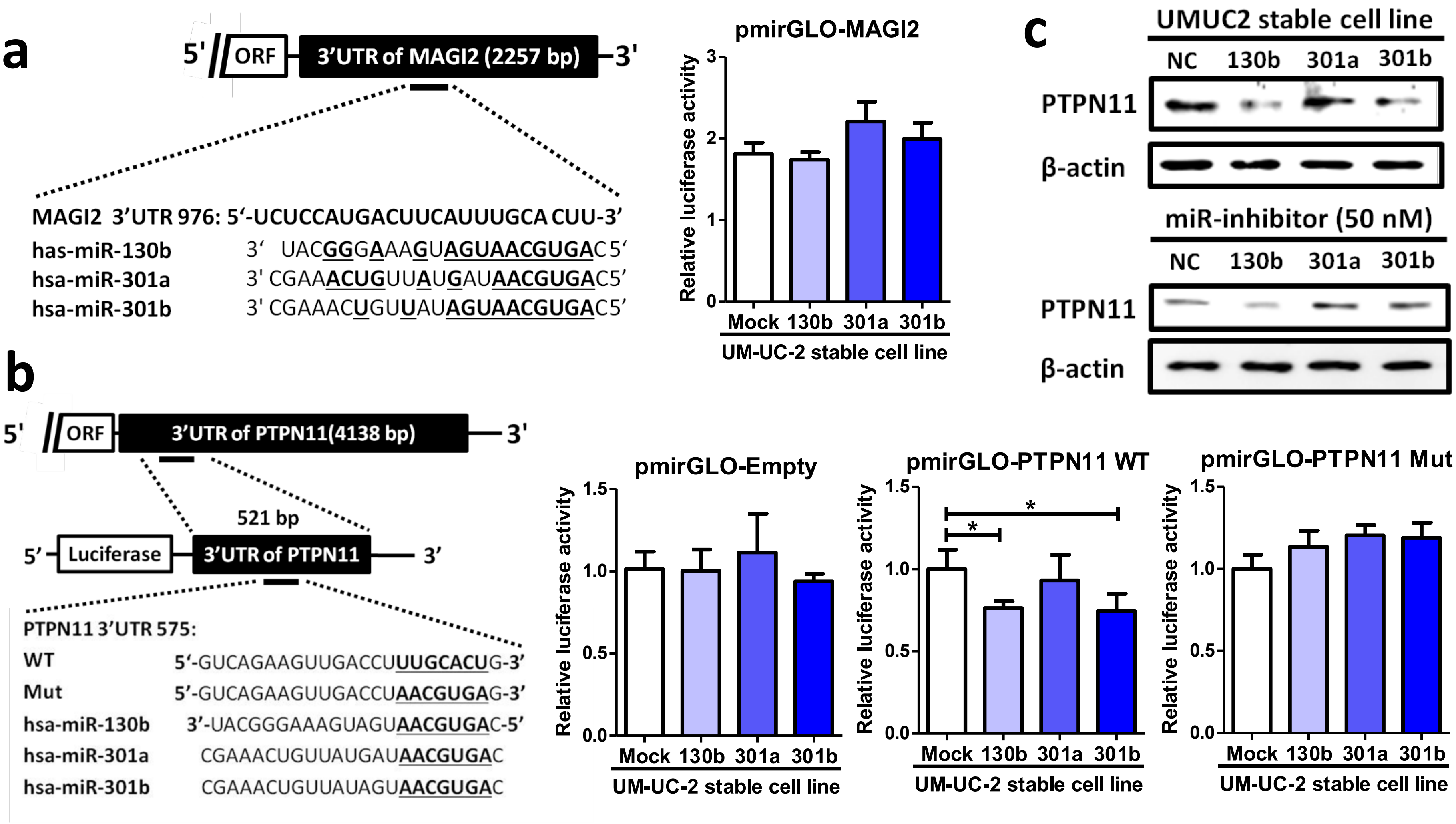

Supplement: Supplementary Information [file srep20574-s1.pdf]
